# State of Inequality in Childhood Immunization: Monitoring Progress Across Low- and Middle-Income Countries over the Past Decade

**DOI:** 10.3390/vaccines14040296

**Published:** 2026-03-26

**Authors:** Nicole Bergen, Anne Schlotheuber, Katherine Kirkby, Luisa Arroyave, M. Carolina Danovaro-Holliday, Aluisio J. D. Barros, Ahmad Reza Hosseinpoor

**Affiliations:** 1 Department of Data, Digital Health, Analytics and AI, World Health Organization, 1211 Geneva, Switzerland; bergenn@who.int (N.B.); schlotheuberan@who.int (A.S.); kirkbyk@who.int (K.K.); 2 International Center for Equity in Health, Universidade Federal de Pelotas, Pelotas 96020-220, Brazil; larroyave@equidade.org (L.A.); abarros@equidade.org (A.J.D.B.); 3 Department of Immunization, Vaccines and Biologicals, World Health Organization, 1211 Geneva, Switzerland; danovaroc@who.int

**Keywords:** childhood immunization, determinants of health, health equity, sustainable development goals, vaccination coverage, vaccines, within-country inequality, zero-dose

## Abstract

**Background/Objectives**: Sizeable between- and within-country inequalities in childhood immunization impair progress towards the goals set by the global Immunization Agenda 2030 (IA2030) of achieving universal coverage of all persons with essential life-saving vaccines. Monitoring global trends in immunization inequalities helps to identify population subgroups that are less likely to benefit from vaccines and provides evidence for tracking progress on regional and global goals and informing equity-oriented interventions. This paper assesses the state of within-country inequality in childhood immunization across low- and middle-income study countries. **Methods**: Using data from household health surveys, the analysis quantifies within-country inequality across up to 92 countries, areas and territories, for nine childhood immunization indicators (seven coverage indicators and two indicators of non-receipt of vaccines) by five dimensions of inequality (child sex, mother’s age, mother’s education, household economic status and place of residence). Absolute and relative summary measures of inequality (difference, ratio, slope index of inequality, relative index of inequality and population attributable risk) were calculated to assess the latest situation of inequality (i.e., using the most recent survey from 2014 to 2023) and change over time (i.e., comparisons with data from 2004 to 2013). **Results**: The latest situation of inequality revealed overall low or no inequality by child sex, mother’s age and place of residence, with more pronounced inequality related to mother’s education and household economic status. The median differences between the most and least educated subgroups ranged between 9 and 14 percentage points for immunization coverage indicators, and between 6 and 9 percentage points for non-receipt of vaccines indicators. The extent of inequality in childhood immunization tended to remain about the same as the previous decade, with modest reductions in absolute economic-related and place of residence inequality in DTP3 immunization, as well as place of residence inequality in full immunization (declining by 3.25, 2.42, and 2.16 percentage points over 10 years, respectively). Distinct patterns of economic-related inequality were evident across country income groups, with low-income countries reporting larger inequality than lower- and upper-middle-income countries; there was substantial variation at the country level. **Conclusions**: Economic- and education-related inequalities in childhood immunization within low- and middle-income countries have persisted over the past decade.

## 1. Introduction

Vaccines are among the most effective and cost-efficient public health interventions, saving millions of lives every year. Since the launch of the Expanded Programme on Immunization in 1974, an estimated 154 million deaths have been averted due to immunization (including 101 million deaths averted in infants younger than 1 year), accounting for 40% of the observed decline in global infant mortality [1]. After experiencing setbacks during the COVID-19 pandemic, many countries are progressing towards restoring childhood immunization coverage to pre-pandemic levels [2], with Gavi, the Vaccine Alliance (Gavi), that serves mainly low-income countries, having reached a record 72 million children through routine immunization programs in 2024 [3]. Marked inequalities in immunization coverage exist both between and within countries, with certain populations less likely to benefit from vaccines. Sustainable Development Goal 3.8, to achieve universal health coverage, includes the aim of ensuring access to vaccines for all [4], which is echoed in Immunization Agenda 2030 (IA2030) [5] and successive Gavi strategies [6].

Quantifying inequalities in childhood immunization and making them “visible” is a vital part of ensuring that programs and interventions accurately identify and reach under-vaccinated children [7]. There are wide variations in the national levels of childhood immunization indicators across countries, as evident in the 2024 WHO/UNICEF (WUENIC) national coverage estimates (see Figure S1 in Supplementary file S1) [8].

National averages can mask substantial inequalities within countries. While immunization programs often emphasize the measurement of spatial inequalities [9], a growing body of research across different settings has characterized within-country inequalities related to socioeconomic, demographic and geographic dimensions [10]. There is considerable variation across studies in terms of the immunization and inequality dimension variables, as well as their approaches to sourcing data, analyzing inequalities and reporting results [10].

Over the past five years (2020–2025), a handful of multi-country studies of within-country inequality in childhood immunization have reported patterns of inequality across childhood immunization indicators by multiple inequality dimensions. An analysis of childhood immunization patterns across 375,000 children in 85 low- and middle-income countries found that the prevalence of zero dose (that is, non-receipt of any doses of diphtheria–pertussis–tetanus (DTP) vaccine) was higher in children with more siblings and higher birth order [11]. Across 64 low- and middle-income study countries, ethnicity-related inequalities in zero-dose prevalence were reported to be statistically significant in more than half of the countries [12]. Children belonging to the majority ethnic group had 29% lower prevalence of zero-dose than the rest of the population [12]. Women’s empowerment (defined based on social independence, decision-making and attitude towards violence) was associated with lower zero-dose prevalence in a study of 50 countries, based on data from Demographic and Health Surveys (DHS) [13]. In 42 countries, the lowest zero-dose prevalence was reported in the groups with the highest level of empowerment [13]. A study of paternal factors on childhood immunization status in African countries reported that children of fathers with primary education or higher were more than three times likely to be fully immunized than those whose fathers had no education, and that joint parental decision-making was linked to higher coverage [14]. A study of 43 low- and middle-income countries explored within-country socioeconomic inequalities in childhood immunization, revealing patterns of wealth-related inequality, and identifying regions with large gaps in coverage (namely, West and Central Africa and South Asia) [15]. Jean Simon et al. assessed inequalities in various childhood immunization indicators across 34 African countries [16]. Factors such as high birth order, low access to antenatal care, poor households and low levels of maternal education were associated with lower levels of full vaccination [16]. Also focused on African countries, Bobo et al. examined immunization inequalities related to indicators of the mother’s socioeconomic position (that is, household wealth, education level and occupation) [17]. Pro-rich inequalities in full vaccination coverage were reported across 23 of the 25 study countries [17]. A study including 38 African countries used data from 2000 to 2019 to estimate immunization coverage inequalities by socioeconomic status and region for five vaccine indicators, and provide projections to 2030. The results suggested that, while immunization inequalities were projected to stabilize or narrow in some settings, others faced increasing inequalities [18].

Among these recent studies, however, there has not been a comprehensive update of childhood immunization inequality that includes countries across all world regions, considers multiple vaccine types and assesses a diverse selection of dimensions of inequality.

Over the past decade, WHO has produced a series of global analyses of inequalities in immunization, along with resources to support capacity strengthening, disaggregated data access and knowledge translation [19]. This includes a global report, published in 2016, that interrogated the state of inequality in five childhood immunization indicators across 69 low- and middle-income countries, considering four dimensions of inequality (household economic status, mother’s education, place of residence and child sex) [20]. The report revealed that national childhood immunization coverage had increased over the previous decade and, although economic- and education-related gaps tended to have narrowed, large inequalities persisted in many countries [20].

This paper aims to provide a global update on the state of within-country inequality in childhood immunization. Motivated by the findings of the 2016 WHO state of inequality report, the present analysis covers a more recent time period, a larger number of low- and middle-income countries, four more childhood immunization indicators and one additional dimension of inequality. The main objectives include: (1) assessing the latest situation of inequality in childhood immunization indicators; (2) making comparisons across countries (benchmarking); (3) presenting data about changes in inequality over the past decade; and (4) showcasing the potential impact of eliminating inequality. Our analysis includes seven immunization coverage indicators and two indicators of non-receipt of vaccines, and five dimensions of inequality (child sex, mother’s age, mother’s education, household economic status and place of residence).

## 2. Materials and Methods

### 2.1. Data Sources

This study is a secondary analysis of data from 182 household surveys, including 96 Demographic and Health Surveys (DHS), 79 Multiple Indicator Cluster Surveys (MICS), 3 Reproductive and Health Surveys (RHS) and 4 non-standard national health surveys (NSS). These are large, nationally representative household health survey programs that collect data about health and sociodemographic variables through standardized face-to-face interviews with women aged 15–49 years. They are conducted in a large number of countries, areas and territories (predominantly low- and middle-income) and repeated approximately every 3 to 5 years. The data in this analysis are the product of a reanalysis of survey microdata by the International Center for Equity in Health (www.equidade.org (accessed on 2 March 2026)) at the Federal University of Pelotas and are publicly available on the WHO Health Inequality Data Repository [21].

### 2.2. Study Countries

Our analysis covered all low- and middle-income settings (countries, areas and territories) in the childhood immunization dataset in the WHO Health Inequality Data Repository with data from a survey conducted between 2014 and 2023. Country income groupings were based on the World Bank classifications for the 2025–2026 fiscal year [22]. One country that was not classified by the World Bank (Ethiopia) was excluded from the analysis, as it could not be assigned to a country income group. In addition, in accordance with the scope of the study on low- and middle-income settings, three high-income countries were excluded.

The analysis includes up to 92 countries, areas and territories, though the number of settings with data for a given indicator–dimension of inequality combination varied. Some surveys did not cover all the immunization indicators in our analysis. This was especially the case with rotavirus, as the vaccine was not universally recommended for inclusion in national immunization programs by the WHO until 2009 [23]. Countries were excluded from the analysis for a given indicator–dimension if estimates were not available for all subgroups (e.g., due to instances of low response for certain subgroups). Data were downloaded from the Health Inequality Data Repository in December 2025. See Table S1 in Supplementary file S1 for a list of study countries, areas and territories, along with corresponding survey sources and years.

### 2.3. Indicators and Dimensions of Inequality

Data about the receipt of childhood vaccines are collected by DHS, MICS, RHS and NSS. Our analysis covers seven indicators of immunization coverage and two indicators that reflect non-receipt of vaccines. The indicators reflect essential vaccines recommended by the WHO Expanded Programme on Immunization for children aged 1 year: one dose of Bacillus Calmette Guérin (BCG) vaccine, three doses of diphtheria–tetanus–pertussis (DTP3) vaccine, three doses of *Haemophilus influenzae* type b (Hib3) vaccine, one dose of measles vaccine, three doses of polio vaccine and last dose of rotavirus vaccine (either second or third dose, depending on the vaccine formulation) [24]. The full immunization indicator used in our analysis captures the receipt of BCG (one dose), DTP3, measles (one dose) and polio (three doses) vaccines. The two indicators of non-receipt of vaccines pertain to the non-receipt of any doses of the DTP vaccine (“zero-dose”) as per the IA2030 definition, and the non-receipt of any doses of the BCG, DTP, measles and polio vaccines [25]. All indicators capture the percentage of children aged 12–23 months who have (or have not) received the specified vaccine (or vaccines) in a given year. In certain countries, the time period of 12–23 months was adjusted to align with the recommended timing for vaccination by national immunization calendars, notably for measles-containing vaccines (18–29 months or 15–26 months). Detailed definitions of all indicators are available on the corresponding metadata page of the WHO Health Inequality Data Repository [26].

Our analysis covers five dimensions of inequality: child sex, mother’s age, mother’s education, household economic status and place of residence. Child sex consists of female and male. Mother’s age consists of two subgroups: 15–19 years and 20–49 years. Mother’s education is grouped into three subgroups: no education, primary education and secondary or higher education. Economic status is based on a household wealth index. Wealth quintiles ranging from quintile 1 (poorest) to quintile 5 (richest) were generated via principal component analysis. Place of residence consists of urban or rural (based on each country’s classification).

### 2.4. Analysis Approach

Our analysis of the latest situation of inequality was based on the latest available data from 2014 to 2023. The change in inequality over time analysis included countries with data available from two time points (2004–2013 and 2014–2023), approximately 10 years apart. When multiple surveys were available, the older survey that fell closest to 10 years prior to the most recent survey was selected (allowing for a minimum of 5 years and a maximum of 15). In some cases, two different surveys were used for the older time period, depending on the availability of data for the immunization indicator. The rotavirus coverage indicator was excluded from the change over time analysis because the number of countries with available data from an earlier time point was low (i.e., less than 5).

Disaggregated data and summary measures of inequality were used to assess inequality across the nine immunization indicators. Summary measures of inequality, including difference and slope index of inequality (SII) (measures of absolute inequality), ratio and relative index of inequality (RII) (measures of relative inequality) and population attributable risk (PAR) (an impact measure of absolute inequality) were calculated at the country level. For immunization coverage indicators, difference was calculated as the indicator value in the more-advantaged subgroup (i.e., older maternal age, most educated, richest quintile or urban; and in the case of child sex, disregarding advantage/disadvantage, male) minus the indicator value in the corresponding less-advantaged subgroup (i.e., younger maternal age, least educated, poorest quintile or rural; and in the case of child sex, disregarding advantage/disadvantage, female); ratio was calculated as the value in the more-advantaged subgroup divided by the less-advantaged subgroup. For non-receipt of vaccines indicators, these calculations were reversed; that is, the less-advantaged subgroup minus (for difference) or divided by (for ratio) the more-advantaged subgroup. SII and RII are regression-based measures, weighted by population size, that represent the difference (in the case of SII) or ratio (in the case of RII) in predicted values of an indicator between the most advantaged and most disadvantaged subgroups, taking into account the situation across all subgroups [27]. SII and RII were calculated for inequality related to economic status and education.

For comparisons of change in inequality over time, absolute change in inequality (measured using difference, ratio, SII and RII) over 10 years was calculated. For example, in the case of absolute change in difference over 10 years, for each country, the annual absolute change was calculated as the difference at time 1 (2014–2023) minus the difference at time 0 (2004–2013), divided by the number of intervening years. Given the differing number of years between surveys, this annual absolute change in difference was multiplied by 10 for comparability across countries (reflecting the absolute change in difference over the past decade). This calculation was done in a similar manner for the ratio, SII and RII.

PAR for education-related inequality was calculated as the difference between the estimate for the most educated subgroup and the national average. It reflects the extent to which the national average would change (i.e., increase, in the case of coverage indicators, or decrease, in the case of non-receipt of vaccines indicators) if education-related inequality were eliminated—that is, if all subgroups had the same coverage or level of non-receipt of vaccines as the most educated subgroup.

To facilitate comparisons across countries, we report the median estimates and 95% confidence intervals globally and by country income group. Regional patterns across WHO regions were explored for potential analysis but excluded because of limited data availability in some regions. To illustrate examples of different patterns in how within-country inequalities have changed over time and the potential impact of eliminating inequality at the national level, comparisons are made between countries in the African Region. Compared to other regions, the African Region has the lowest overall coverage of childhood immunization (and therefore the largest opportunity for improvement), and data were available for a large number of countries in the region (i.e., 34 countries).

All analyses were weighted to account for the survey sampling design. We conducted our analysis using STATA version 18.0. In addition, data were explored through interactive data visualizations prepared in Tableau version 2025.2.4.

The paper highlights key findings from the analysis, drawing on DTP3 immunization coverage and DTP (zero-dose) as proxy indicators to illustrate trends observed across other indicators, where applicable. The full results are available for further exploration via interactive data visuals (see: https://public.tableau.com/app/profile/who.inequality.monitor/viz/2026_state_of_inequality_immunization/Cover (accessed on 2 March 2026)).

## 3. Results

### 3.1. Latest Situation: National Average

The total number of study countries with data from 2014 to 2023 surveys ranged from 88 to 92 for all immunization indicators except rotavirus (where data from up to 54 countries were included). For immunization coverage indicators, the median national coverage was lowest for full immunization (63.2%) and highest for BCG (94.7%). For non-receipt of vaccines indicators, the median level of DTP (zero-dose) was 7.0%, whereas the median level of non-receipt of BCG, DTP, measles and polio was 3.7% (Table 1).

### 3.2. Latest Situation: Within-Country Inequality

The number of countries included in the latest situation of inequality analysis was lowest for the mother’s age dimension of inequality (41–42 countries for all indicators except rotavirus, which covered 30 countries) and highest for the economic status, place of residence and child sex dimensions (87–92 countries for indicators except rotavirus, which covered 54 countries). The number of countries with available data disaggregated by mother’s education level ranged from 66 to 68 across all indicators except rotavirus (covering 43 countries) (see Table S2 in Supplementary file S1).

Overall inequalities by mother’s age tended to be small in terms of both absolute and relative inequalities, and there was no overall inequality between females and males. Similarly, there was no overall inequality related to place of residence, though the situation differed across countries, with some countries reporting higher coverage among urban areas and others among rural areas. Within-country absolute and relative inequalities related to household economic status and mother’s education level tended to be more pronounced (see Table S2 in Supplementary file S1).

**Figure 1:**
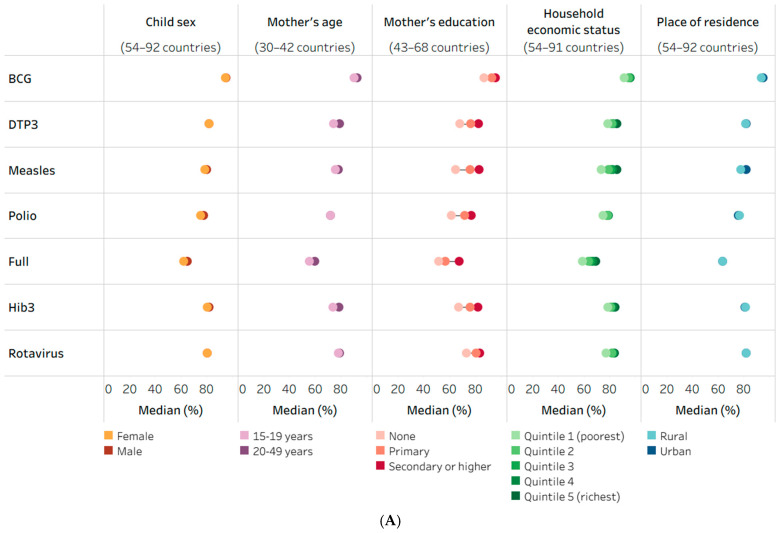

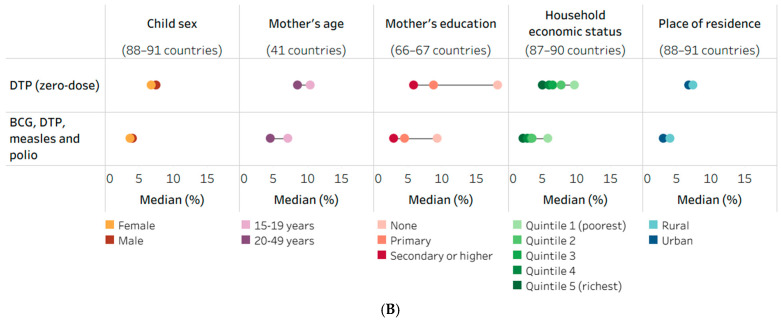
Median estimates of childhood immunization indicators across low- and middle-income countries, disaggregated by child sex, mother’s age, mother’s education, household economic status and place of residence, 2014–2023 DHS, MICS and NSS. (**A**) Immunization coverage among one-year-olds (%). (**B**) Prevalence of non-receipt of vaccines among one-year-olds (%). BCG: Bacillus Calmette–Guérin; DHS: Demographic and Health Surveys; DTP3: three doses of diphtheria–tetanus–pertussis vaccine; Hib3: three doses of *Haemophilus influenzae* type b vaccine; MICS: Multiple Indicator Cluster Surveys; NSS: non-standard national health surveys. Polio indicator reflects the receipt of three doses of any polio vaccine; rotavirus indicator reflects the receipt of the last dose. Full includes BCG (one dose), DTP3, measles (one dose) and polio (three doses). Each circle represents the median estimate for the corresponding indicator and subgroup across study countries; horizontal lines represent the range between the highest and lowest subgroup estimates. For country numbers by indicator and inequality dimension, see Table S2 in Supplementary file S1.

Figure 1 illustrates the median coverage of immunization indicators by subgroups. Data for maternal age indicated slightly higher coverage among the 20–49-year subgroup compared to the 15–19-year subgroup for most coverage indicators, and no apparent patterns of advantage or disadvantage were observed by place of residence. Similarly, there were almost no differences in the median coverage levels between male and female children for most coverage indicators (Figure 1A).

More pronounced subgroup differences were evident for the economic status and education dimensions. The median level of coverage across countries was consistently higher in the richest compared to the poorest quintile for all coverage indicators, with variable patterns observed across quintiles 2–4. Inequalities in median coverage by the mother’s education showed distinct patterns of increasing coverage with increasing education levels. For the BCG and rotavirus indicators, the median levels of coverage in the two most educated subgroups were similar (within 3.0 percentage points) and markedly higher than in the least educated subgroup (Figure 1A).

The non-receipt of vaccines indicators demonstrated similar patterns of advantage and disadvantage across subgroups. Across study countries, we found that the median prevalence of non-receipt of BCG, DTP, measles and polio was 9.3% among the least educated group (versus 3.0% in the most educated subgroup), 7.1% among the group of younger mothers (versus 4.5% in the older subgroup) and 5.8% among the poorest households (versus 2.2% in the richest subgroup) (Figure 1B).

**Figure 2:**
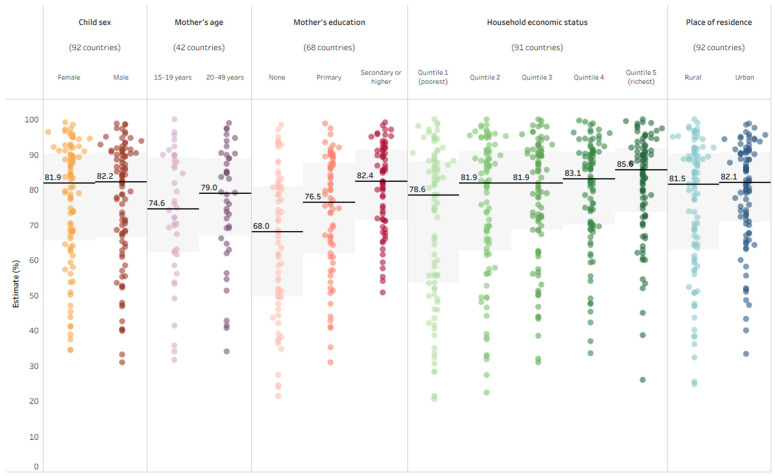
DTP3 immunization coverage among one-year-olds (%), disaggregated by child sex, mother’s age, mother’s education, household economic status and place of residence, 2014–2023 DHS, MICS and NSS. DHS: Demographic and Health Surveys; DTP3: three doses of diphtheria–tetanus–pertussis vaccine; MICS: Multiple Indicator Cluster Surveys; NSS: non-standard national health surveys. In each column, every study country is represented by one circle showing the subgroup estimate; horizontal lines indicate the median estimate across study countries for the corresponding subgroup; gray shading indicates the interquartile range (middle 50% of country estimates).

The levels of coverage for the indicator–dimension subgroup combinations varied across study countries. Figure 2 shows the range of country estimates for DTP3 immunization coverage across subgroups. For nearly all subgroups, the range of values across countries spans around 65 percentage points or higher. For example, the coverage of DTP3 immunization in one-year-olds of the poorest households (quintile 1) ranged from 20.6% in the Central African Republic in 2019 to 100.0% in Turkmenistan in 2015. The subgroup corresponding to the mother’s education level of secondary or higher education was an exception, as the range of estimates spanned less than 50 percentage points, from 50.8% (in the Democratic Republic of Congo, 2017) to 99.3% (in Rwanda, 2019). Compared to the DTP3 indicator, the range of country estimates tended to be narrower for BCG and measles coverage indicators and for the two non-receipt of vaccines indicators.

DTP3 immunization coverage displayed a gradient pattern across subgroups for household economic status and mother’s education. In the case of economic status, the median level of coverage was about the same in quintiles 2, 3 and 4, with lower coverage among the poorest and higher coverage among the richest. Across the three education subgroups, step-wise increases were observed with increasing levels of education. While the median levels of coverage were about the same across rural and urban areas, there was variation between countries, with several countries reporting an absolute difference of 10 percentage points or more between urban and rural areas, favoring either urban (n = 21) or rural (n = 4) areas. Overall, there was no difference between median values of female and male children; however, a difference of 4.4 percentage points was reported between median values of children of mothers aged 20–49 years and those of mothers aged 15–19 years.

**Figure 3:**
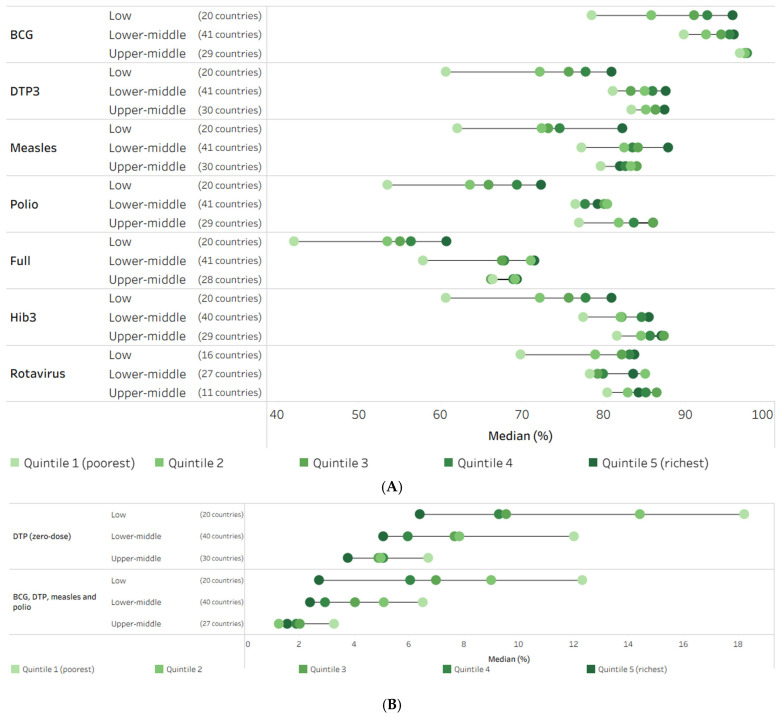
Median estimates of childhood immunization indicators by country income group, disaggregated by household economic status, 2014–2023 DHS, MICS and NSS. (**A**) Immunization coverage among one-year-olds (%) (**B**) Prevalence of non-receipt of vaccines among one-year-olds (%). BCG: Bacillus Calmette–Guérin; DHS: Demographic and Health Surveys; DTP3: three doses of diphtheria–tetanus–pertussis vaccine; Hib3: three doses of *Haemophilus influenzae* type b vaccine; MICS: Multiple Indicator Cluster Surveys; NSS: non-standard national health surveys. Polio indicator reflects the receipt of three doses of any polio vaccine; rotavirus indicator reflects the receipt of the last dose. Full includes BCG (one dose), DTP3, measles (one dose) and polio (three doses). Each circle represents the median estimate across study countries for the corresponding indicator, World Bank income group and subgroup; horizontal lines represent the range between the highest and lowest subgroup estimates.

Across country income groupings, there was variation in the median level of immunization indicators in subgroups. For example, overall economic-related inequality was larger in low-income countries compared to lower-middle- and upper-middle-income countries for all immunization indicators (Figure 3). The poorest quintile of the low-income country group demonstrated substantially lower coverage compared to the other four quintiles for DTP3, measles, polio, full, Hib3 and rotavirus immunization coverage (Figure 3A).

The two non-receipt of vaccines indicators demonstrated higher median prevalence of non-receipt of vaccines among the poorest versus richest quintiles, especially in low-income study countries (Figure 3B). The gap between the median prevalence in the poorest and richest subgroups reached 11.8 percentage points for zero-dose (DTP) in 20 low-income study countries. For further exploration of childhood immunization data by country income group, disaggregated by other dimensions of inequality, see https://public.tableau.com/app/profile/who.inequality.monitor/viz/2026_state_of_inequality_immunization/Cover (accessed on 2 March 2026).

### 3.3. Change over Time: Within-Country Inequality

**Figure 4:**
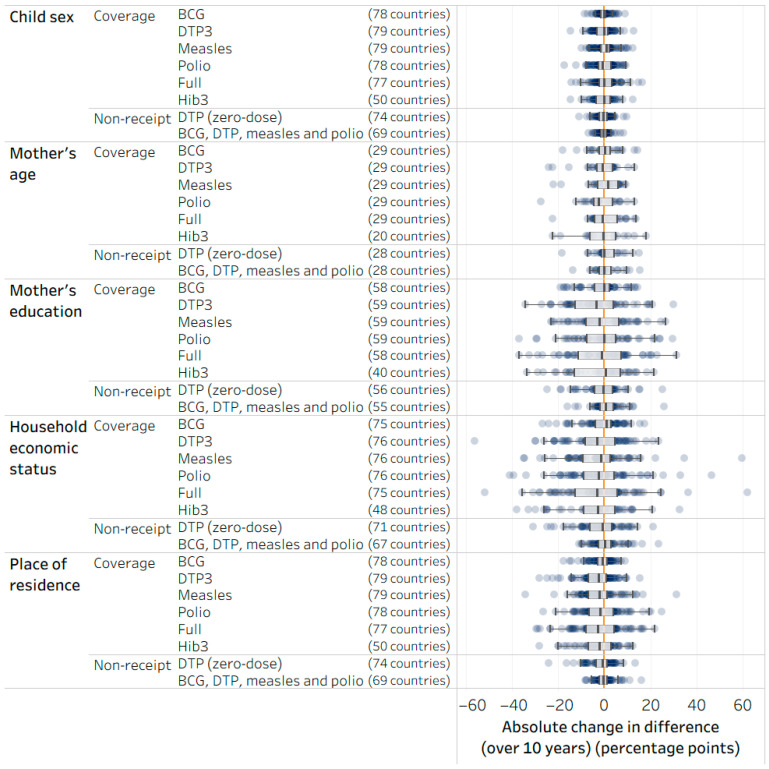
Absolute change in difference over 10 years for childhood immunization indicators, by child sex, mother’s age, mother’s education, household economic status and place of residence, 2004–2013 and 2014–2023, DHS, MICS, RHS and NSS. BCG: Bacillus Calmette–Guérin; DHS: Demographic and Health Surveys; DTP3: three doses of diphtheria–tetanus–pertussis vaccine; Hib3: three doses of *Haemophilus influenzae* type b vaccine; MICS: Multiple Indicator Cluster Surveys; NSS: non-standard national health surveys; RHS: Reproductive and Health Surveys. Polio indicator reflects the receipt of three doses of any polio vaccine. Full includes BCG (one dose), DTP3, measles (one dose) and polio (three doses). In each row, every study country is represented by one circle showing the absolute change in difference over 10 years; box plots represent the median estimate (middle line), middle 50% of values (center gray box) and remaining 25% of upper and lower values (outer lines), excluding outliers across study countries for the corresponding inequality dimension and indicator. The orange vertical line indicates the value of no change over time (zero).

The number of countries included in the change in within-country inequality over time analyses ranged from 20 (for Hib immunization by age) to 79 (for DTP3 and measles by place of residence and sex). Overall, inequalities in the eight childhood immunization indicators tended to remain about the same over the previous decade. The absolute change in difference over 10 years is shown in Figure 4. There were small reductions in overall levels of absolute inequality in a few cases, such as economic-related and place of residence inequality in DTP3 and place of residence inequality in measles immunization (declining by an average of 3.3, 2.4 and 2.2 percentage points (over 10 years), respectively) (see Table S3 in Supplementary file S1).

**Figure 5:**
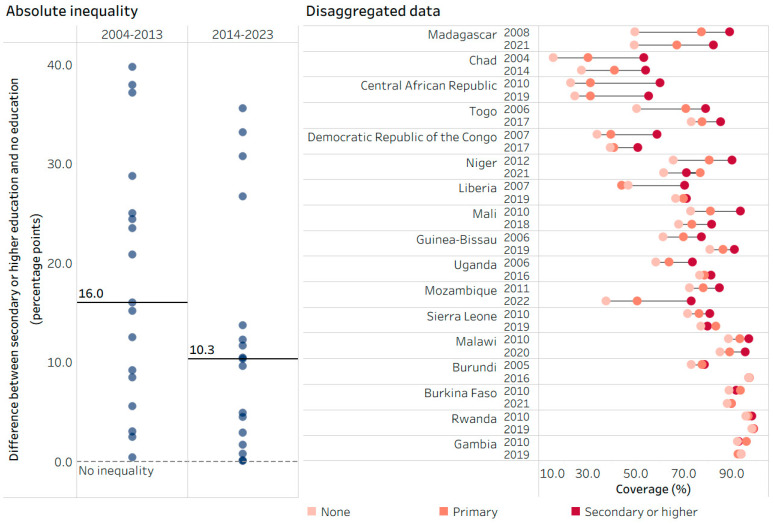
DTP3 immunization coverage among one-year-olds (%): median estimates of absolute education-related inequality and country-level disaggregated data by mother’s education level, 17 low-income countries in the WHO African Region, 2004–2013 and 2014–2023, DHS and MICS. DHS: Demographic and Health Surveys; DTP3: three doses of diphtheria–tetanus–pertussis vaccine; MICS: Multiple Indicator Cluster Surveys. On the left, each study country is represented by two circles (one for each time point) showing the level of absolute education-related inequality (difference between secondary or higher education and no education); the horizontal lines represent the median difference at each time point. On the right, each circle represents the subgroup estimate in the corresponding country and survey year; horizontal lines represent the range between the highest and lowest subgroup estimates for each country and survey year. Countries are sorted from highest to lowest difference in time 0 (2004–2013).

Country-level variations in patterns of inequality over time were apparent. For instance, across 17 low-income countries in the WHO African Region, the median education-related inequality in DTP3 coverage (calculated as the difference between the most and least educated subgroups) declined from 16.0 percentage points in 2004–2013 to 10.3 percentage points in 2014–2023 (Figure 5). In countries such as Burundi, Chad, Guinea-Bissau, Liberia, Togo and Uganda, education-related inequality decreased over the previous decade with notable gains in coverage among the two least educated subgroups. In Burkina Faso, Gambia and Rwanda, DTP3 coverage remained above 85% in all subgroups across the two time points, while Mozambique reported declines in coverage in all subgroups, with widening inequality. For further exploration of the change over time in childhood immunization indicators, see https://public.tableau.com/app/profile/who.inequality.monitor/viz/2026_state_of_inequality_immunization/Cover (accessed on 2 March 2026).

### 3.4. Impact of Eliminating Education-Related Inequality

**Figure 6:**
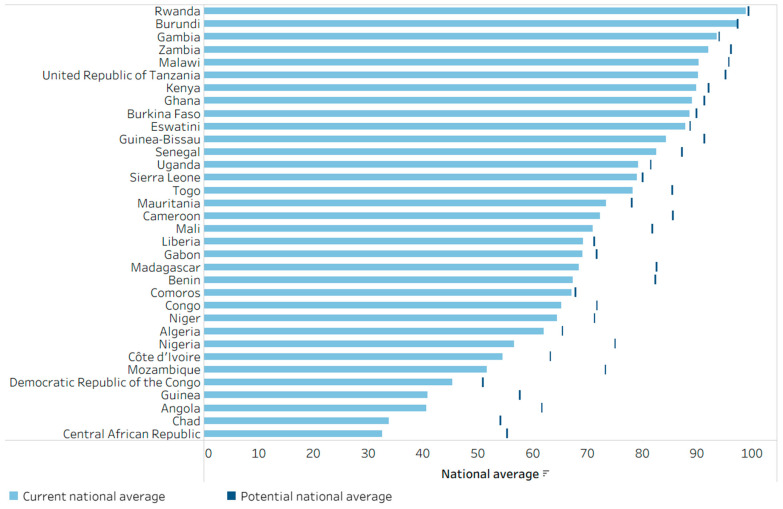
Potential for improvement in the national average of DTP3 immunization coverage among one-year-olds by eliminating inequality related to the mother’s education, 34 countries in the WHO African Region, 2014–2023, DHS and MICS. DHS: Demographic and Health Surveys; DTP3: three doses of diphtheria–tetanus–pertussis vaccine; MICS: Multiple Indicator Cluster Surveys. The potential national average (vertical dark blue line) reflects the national average that could be achieved if all one-year-olds had the same level of coverage as the most-educated subgroup. Countries are sorted from highest to lowest current national average (light blue bar).

The summary measure PAR was calculated for the mother’s education to demonstrate the potential impact of eliminating inequality. Figure 6 shows the projected level of national DTP3 immunization coverage across 34 African countries if all subgroups had the same coverage as the most educated (based on the latest available data). In Burundi, Gambia and Rwanda, where education-related inequalities were low and national coverage high, there was little room for improvement. However, countries with large education-related inequality and low national coverage demonstrated a large potential for improvement. In Angola, Central African Republic, Chad and Mozambique, for instance, national DTP3 coverage would increase by more than 20 percentage points if all subgroups had the same level of coverage as the most-educated subgroup. Across this group of 34 African countries, 10 countries reported a potential for improvement of at least 10 percentage points.

**Figure 7:**
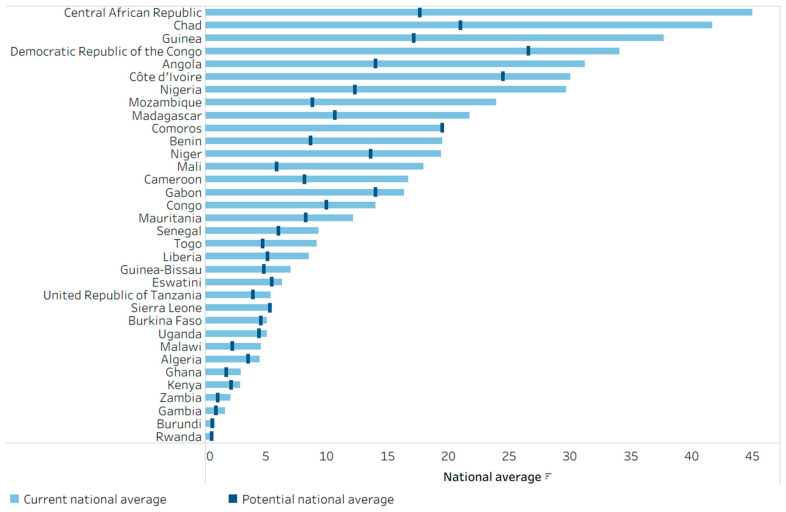
Potential for improvement in zero-dose DTP prevalence among one-year-olds by eliminating inequality related to the mother’s education, 34 countries in the WHO African Region, 2014–2023 DHS and MICS. DHS: Demographic and Health Surveys; DTP: diphtheria–tetanus–pertussis vaccine; MICS: Multiple Indicator Cluster Surveys. The potential national average (vertical dark blue line) reflects the national average that could be achieved if all one-year-olds had the same zero-dose prevalence as the most-educated subgroup. Countries are sorted from highest to lowest current national average (light blue bar).

Figure 7 shows the analogous PAR calculation for the zero-dose DTP indicator (the extent to which the national average of zero-dose DTP prevalence would decline if all subgroups matched the prevalence in the most-educated subgroup). Across 34 African countries, 17 reported less than 10% national zero-dose DTP prevalence based on the latest survey. By eliminating education-related inequality, an additional 6 countries would drop to less than 10% zero-dose DTP prevalence. In 14 countries, zero-dose prevalence would be at least halved if education-related inequality were eliminated (Angola, Benin, Cameroon, Central African Republic, Chad, Gambia, Guinea, Madagascar, Malawi, Mali, Mozambique, Nigeria, Togo, and Zambia).

## 4. Discussion

Using data from multinational household surveys, our analysis provides an update on the state of inequality in childhood immunization indicators across 92 low- and middle-income countries, areas and territories. The present analysis is an extension of a report published by WHO nearly 10 years ago, which drew attention to widespread and persistent within-country inequalities in childhood immunization [20].

Similar to the WHO inequality analysis conducted 10 years ago [20], and other more recent analyses [15,17,28], there is evidence of sustained between-country inequality across childhood immunization indicators. Although efforts by organizations such as Gavi, the Vaccine Alliance, have scaled up efforts to provide childhood vaccines in priority countries, backsliding during COVID-19 [29,30] along with both demand-side barriers (such as beliefs, intentions and behaviors [31,32,33,34]) and supply-side barriers (such as facility readiness and financing [34,35]) are some of the factors that account for the lack of widespread improvement across all countries.

Overall, within-country inequalities showed little change in inequality over the past decade. They were most pronounced for the mother’s education and household economic status, whereby more educated and richer subgroups were generally advantaged (that is, reported higher coverage and/or lower prevalence of non-receipt of vaccines). There were small or no inequalities overall by mother’s age, place of residence and child sex, though our analysis provided some evidence of divergent patterns of place of residence inequality at the country level. These findings mirror trends reported in the WHO 2016 analysis of larger inequalities related to economic status and education, and smaller inequalities in terms of sex and place of residence [20], though a decade ago, there was evidence that economic- and education-related inequalities in childhood immunization were narrowing.

Inadequate immunization has been associated with younger maternal age and lower levels of maternal education in previous studies [16,17,36,37]. Possible explanations include access to health services, awareness and knowledge about vaccines, and attitudes towards vaccination, though there has been little research devoted to vaccine decision-making among mothers in low- and middle-income country contexts [38,39]. A better understanding of maternal barriers and enablers would provide valuable evidence to inform targeted strategies to improve immunization coverage. Our findings also support the further study of the role of women’s empowerment in relation to childhood immunization [13,40,41].

**Figure 8:**
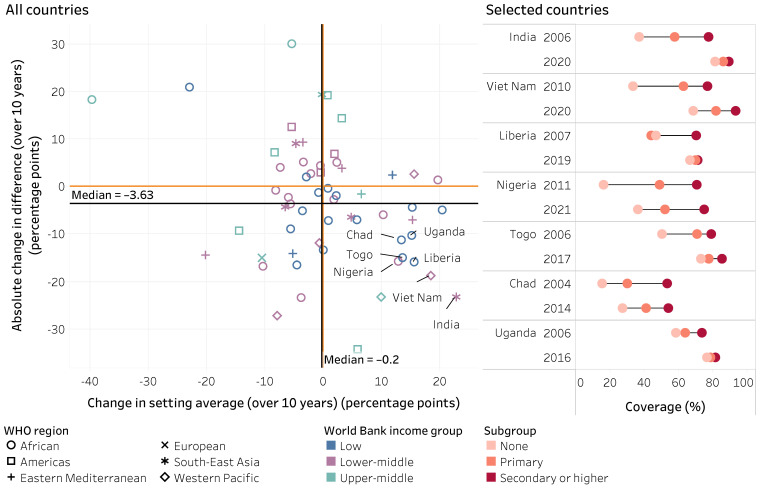
Absolute change in difference and setting average over 10 years for DTP3 coverage among one-year-olds, by mother’s education, 59 countries, 2004–2013 and 2014–2023, DHS, MICS, RHS and NSS. DHS: Demographic and Health Surveys; DTP3: three doses of diphtheria–tetanus–pertussis vaccine; MICS: Multiple Indicator Cluster Surveys; NSS: non-standard national health surveys; RHS: Reproductive and Health Surveys. On the left, each study country is represented by one shape; the black lines represent the median value across study countries; the orange lines indicate the value of no change (zero). On the right, each circle represents the subgroup estimate in the corresponding country and survey year; horizontal lines represent the range between the highest and lowest subgroup estimates. Countries are sorted from highest to lowest absolute change in difference (over 10 years).

**Figure 9:**
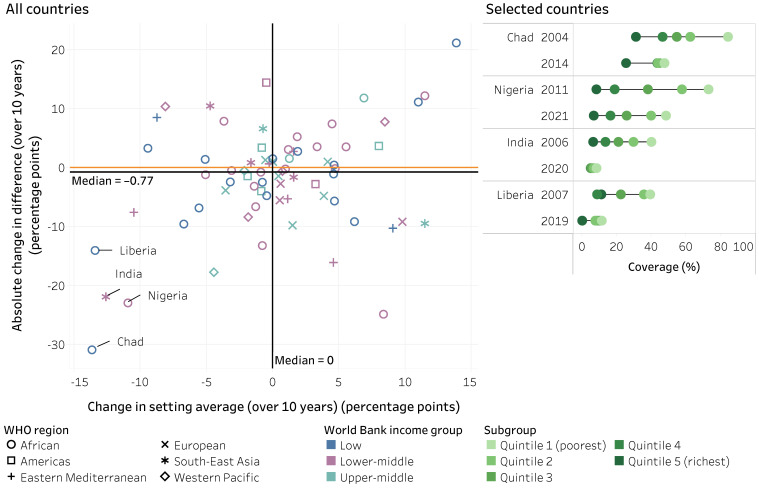
Absolute change in difference and setting average over 10 years for zero-dose DTP prevalence among one-year-olds, by household economic status, 71 countries, 2004–2013 and 2014–2023, DHS and MICS. DHS: Demographic and Health Surveys; DTP: diphtheria–tetanus–pertussis vaccine; MICS: Multiple Indicator Cluster Surveys. On the left, each study country is represented by one shape; the black lines represent the median value across study countries; the orange lines indicate the value of no change (zero). On the right, each circle represents the subgroup estimate in the corresponding country and survey year; horizontal lines represent the range between the highest and lowest subgroup estimates. Countries are sorted from highest to lowest absolute change in difference (over 10 years).

At the country level, there was wide variation in the level of immunization indicators by subgroups, and countries demonstrated distinct patterns of change in inequality over time. This variation was observed across country-income groupings and WHO regions, as illustrated in our analysis of education-related inequality in DTP coverage across 17 low-income countries in the WHO African Region (where countries reported improving and worsening situations). Across countries, variable patterns of change were observed across immunization indicators and dimensions of inequality over the 10-year study period. For example, the following figures illustrate the absolute changes in difference versus the change in national averages for education-related inequality in DTP3 coverage (Figure 8) and economic-related inequality in non-receipt of DTP (zero-dose) (Figure 9). Data from the countries with the most substantial reductions in inequality alongside improvements in national averages are displayed in more detail on the right of each figure. More research is warranted to explore the factors that contribute to these changes within these countries (noting that other, high-performing countries may have reported little improvement due to having sustained low inequality alongside high coverage over the study period).

Our analysis of the potential impact of eliminating education-related inequality also illustrated wide variations across countries. These findings affirm the importance of specific planning and target setting at national levels. Equity is a key strategic priority in IA2030, which acknowledges that strategies and interventions known to improve equity are often underutilized [42]. A systematic review of pro-equity strategies across high-, middle- and low-income countries found that the majority of interventions focused on improving immunization coverage in priority groups address acceptability (65% of studies), contact (37% of studies) or accessibility (31% of studies) [43]. Another study, focused on low socioeconomic status groups within developed countries, found comprehensive multi-component interventions to be most effective, including those attending to improving access, appointment reminders, education and tailored health communications [44]. In 2025, WHO and UNICEF called on governments and development partners to address immunization inequality through: strengthening immunization in conflict and fragile settings; embedding immunization within primary health care systems; countering misinformation; and investing in strong data and disease surveillance systems [45].

Across country income groups, the overall level of economic-related inequality was uniformly higher in low-income countries than in lower-middle- and upper-middle-income countries. A study of childhood immunization coverage in 195 countries reported declines in routine childhood vaccine coverage during the COVID-19 pandemic that disproportionately affected children in low-income countries [29]. Low-income settings face unique challenges in progressing on equity-focused approaches, which may include resource limitations, weak governmental institutions, rudimentary or damaged health infrastructure, high levels of health needs and less functional health information systems [27].

### Strengths, Limitations and Methodological Considerations

A strength of this study was our inclusion of 92 countries across low-, lower-middle- and upper-middle-income country groupings. The use of similar analysis approaches enabled the comparison of our results with those published by WHO in 2016 [20]. Our inclusion of two non-receipt of vaccines indicators offered additional insights into childhood immunization inequalities. Identifying and characterizing zero-dose children is a core priority for IA2030 and Gavi, as they represent populations who lack access to essential health services [25]. Our analysis revealed a lower prevalence of non-receipt of vaccines in the 20–49-year subgroup than in the 15–19-year subgroup, and among the richer and more educated subgroups, compared to their respective counterparts.

Our study made use of data from prominent global household survey programs, including DHS (administered by the United States Agency for International Development) and MICS (administered by UNICEF). These programs provide similar data across many low- and middle-income countries and are a main source of disaggregated data for assessing within-country inequality in childhood immunization in many low- and middle-income countries [10,46]. The data, however, are subject to recall bias, particularly in countries where the availability of vaccination cards seen is low [47], as well as other sources of non-random errors; surveys are also conducted at multi-year intervals, which vary across countries. For this reason, we identified a range of years for our latest situation and change over time analyses, which may affect the comparability of results across countries.

In this study, we relied on both absolute and relative summary measures of inequality to capture the latest situation and change in inequality over time. Previously, Kirkby et al. (2024) demonstrated the importance of employing absolute and relative measurements to capture different aspects of inequality in childhood immunization indicators [48]. In our analysis, the use of ratios highlighted elevated relative inequality in non-receipt of vaccines indicators (see Table S2 in Supplementary file S1). Due to the nature of the indicators, the prevalence estimates of non-receipt of vaccines fell at the lower end of the numerical scaling than the coverage estimates, and thus small differences in prevalence translated into higher levels of relative inequality [27].

The median national childhood immunization values reported in this analysis are from surveys, and thus differ from the 2024 estimates produced by WHO/UNICEF (variably higher, about the same or lower), which triangulate data from various sources [49]. The values reported in our analysis are based on a subset of low- and middle-income countries, reflect a wider range of years, and use different methodologies and data sources [50].

Our methods allowed for a comparison of results with the 2016 WHO report; however, certain analytical decisions may have led to an underestimation of inequality when compared with other studies. For example, we applied a “low resolution” categorization to show geographical inequality according to urban versus rural areas. The use of more granular categories (remote rural, semi-rural, suburban, etc.) may have revealed greater within-country geographical inequality. Increasingly, explorations of geographical inequality are conducted at high levels of spatial resolution. With regard to economic-related inequality, childhood immunization coverage inequalities captured using a multivariate index (based on multiple forms of disadvantage) were found to be 32% to 324% larger than inequalities measured as absolute comparisons between quintiles (as applied in our analysis) [51].

## 5. Conclusions

Inequality monitoring is a crucial step in understanding and addressing inequalities in childhood immunization. The findings of this study point to persistent economic- and education-related inequalities in childhood immunization within low- and middle-income countries over the past decade, indicating that progress in narrowing within-country inequalities (as observed 10 years ago) has largely stagnated. The trends observed within specific countries and country-income groups, however, reveal more varied situations and patterns of change. Realizing the aspiration of the IA2030 goals and achieving universal childhood immunization will require renewed efforts to close remaining coverage gaps. This includes continued and intensified targeting tailored to the poorest and least educated populations in low- and middle-income countries. The results of this study serve as a reference point for future monitoring of childhood immunization inequality.

## Figures and Tables

**Table 1:** National estimates of childhood immunization indicators (%), point estimates, across 54–92 low- and middle-income countries, 2014–2023 DHS, MICS and NSS.

Indicator	Number of Countries	Minimum	25th Percentile	Median (50th Percentile)	75th Percentile	Maximum
Immunization coverage						
BCG	91	59.6	86.7	94.7	97.6	100.0
DTP3	92	32.6	65.9	81.9	90.5	99.0
Measles	92	39.5	64.0	78.9	88.0	97.7
Polio	91	21.2	58.5	76.0	87.3	98.8
Full	90	13.4	44.9	63.2	79.2	95.5
Hib3	90	32.6	64.5	81.3	90.0	99.0
Rotavirus	54	1.0	64.3	80.5	90.2	99.3
Non-receipt of vaccines						
DTP (zero-dose)	91	0.4	3.5	7.0	17.9	45.0
BCG, DTP, measles and polio	88	0.0	1.3	3.7	9.8	38.2

BCG: Bacillus Calmette–Guérin; DHS: Demographic and Health Surveys; DTP3: three doses of diphtheria–tetanus–pertussis vaccine; Hib3: three doses of *Haemophilus influenzae* type b vaccine; MICS: Multiple Indicator Cluster Surveys; NSS: non-standard national health surveys. Polio indicator reflects the receipt of three doses of vaccine; rotavirus indicator reflects the receipt of the last dose. Full includes BCG (one dose), DTP3, measles (one dose) and polio (three doses).

## Data Availability

The data used in this analysis are publicly available through the WHO Health Inequality Data Repository (https://www.who.int/data/inequality-monitor/data (accessed on 2 March 2026)). Datasets were downloaded from the Repository in December 2025. The data presented in this article are additionally available from https://public.tableau.com/app/profile/who.inequality.monitor/viz/2026_state_of_inequality_immunization/Cover (accessed on 2 March 2026).

## Supplementary Materials

The following supporting information can be downloaded at: https://www.mdpi.com/article/10.3390/vaccines14040296/s1: Figure S1. WHO/UNICEF estimates of childhood immunization indicators (%) among one-year-olds, 2024; Table S1. Study countries, areas and territories, survey sources and years, WHO region and country income group; Table S2. Childhood immunization indicator median differences and ratios, by dimensions of inequality, 2014–2023 DHS, MICS and NSS; Table S3. Childhood immunization indicators (%), median differences by dimensions of inequality, 2004–2013 and 2014–2023 DHS, MICS, RHS and NSS. For further exploration of the childhood immunization data presented in this article, see interactive data dashboards, available here: https://public.tableau.com/app/profile/who.inequality.monitor/viz/2026_state_of_inequality_immunization/Cover (accessed 2 March 2026).

## Abbreviations

The following abbreviations are used in this manuscript:

BCG	Bacillus Calmette–Guérin
DHS	Demographic and Health Surveys
DTP	Diphtheria–Tetanus–Pertussis Vaccine
DTP3	Three Doses of Diphtheria–Tetanus–Pertussis Vaccine
Hib3	Three Doses of *Haemophilus influenzae* type b Vaccine
IA2030	Immunization Agenda 2030
MICS	Multiple Indicator Cluster Surveys
NSS	Non-Standard National Health Surveys
PAR	Population Attributable Risk
RHS	Reproductive and Health Surveys
RII	Relative Index of Inequality
SII	Slope Index of Inequality
UNICEF	United Nations Children’s Fund
WHO	World Health Organization
WUENIC	WHO/UNICEF Estimates of National Immunization Coverage
