# Supplementary material for: State of Inequality in Childhood Immunization: Monitoring Progress Across Low- and Middle-Income Countries over the Past Decade
Source: Vaccines (Basel). 2026 Mar 26;14(4):296. doi: 10.3390/vaccines14040296 (PMC13119883; doi:10.3390/vaccines14040296)
Supplement: Supplementary file 1 [file vaccines-14-00296-s001.zip › vaccines-4160568-supplementary.pdf]

Supplementary file S1

Figure S1. WHO/UNICEF estimates of childhood immunization indicators (%) among one-year-olds, 2024

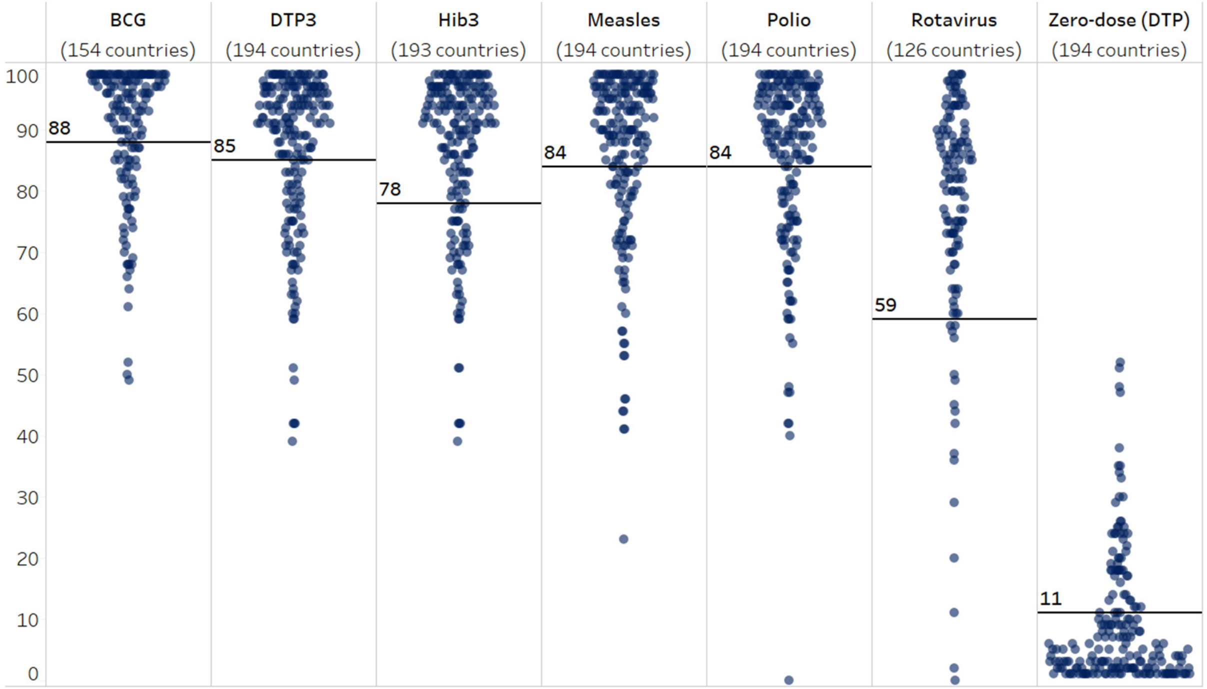

Data source: World Health Organization Immunization Data. Available online: https://immunizationdata.who.int/global (accessed on 17 October 2025).

BCG: Bacillus Calmette–Guérin; DTP3: three doses of diphtheria–tetanus–pertussis vaccine; Hib3: three doses of *Haemophilus influenzae* type b vaccine. Polio indicator reflects the receipt of three doses of any polio vaccine; rotavirus indicator reflects the receipt of the last dose of the series (second or third depending on formulation used). Zero-dose (DTP) reflects the non-receipt of any doses of the DTP vaccine, as per the IA2030 definition of "zero-dose". In each column, each circle represents one country; horizontal lines indicate the median estimates across countries. The N reflects the number of countries that include the vaccine in its immunization schedule and have reported data to WHO and UNICEF at least once.

Table S1. Study countries, areas and territories, survey sources and years, WHO region and country income group

Setting	Survey(s)	WHO Region	World Bank country income group
Afghanistan	MICS 2022, MICS 2011	Eastern Mediterranean	Low-income
Albania	DHS 2017, DHS 2008	European	Upper-middle-income
Algeria	MICS 2019, MICS 2012	African	Upper-middle-income
Angola	DHS 2015	African	Lower-middle-income
Armenia	DHS 2016, DHS 2005	European	Upper-middle-income
Azerbaijan	MICS 2023	European	Upper-middle-income
Bangladesh	DHS 2017, DHS 2007	South-East Asia	Lower-middle-income
Belize	MICS 2016, MICS 2006	Americas	Upper-middle-income
Benin	MICS 2021, DHS 2012	African	Lower-middle-income
Bolivia (Plurinational State of)	NSS 2016, DHS 2008	Americas	Lower-middle-income
Burkina Faso	DHS 2021, DHS 2010	African	Low-income
Burundi	DHS 2016, DHS 2010, MICS 2005	African	Low-income
Cambodia	DHS 2021, DHS 2010	Western Pacific	Lower-middle-income
Cameroon	DHS 2018, MICS 2006	African	Lower-middle-income
Central African Republic	MICS 2019, MICS 2010	African	Low-income
Chad	DHS 2014, DHS 2004	African	Low-income
Comoros	MICS 2022, DHS 2012	African	Lower-middle-income
Congo	MICS 2014, DHS 2005	African	Lower-middle-income
Cuba	MICS 2019, MICS 2010	Americas	Upper-middle-income
Côte d'Ivoire	DHS 2021, DHS 2012	African	Lower-middle-income
Democratic Republic of the Congo	MICS 2017, DHS 2007	African	Low-income
Dominican Republic	MICS 2019, DHS 2007	Americas	Upper-middle-income
Ecuador	NSS 2018, NSS 2012	Americas	Upper-middle-income
Egypt	DHS 2014, DHS 2005	Eastern Mediterranean	Lower-middle-income
El Salvador	MICS 2014, RHS 2008	Americas	Upper-middle-income

Eswatini	MICS 2022, MICS 2010	African	Lower-middle-income
Fiji	MICS 2021	Western Pacific	Upper-middle-income
Gabon	DHS 2019, DHS 2012	African	Upper-middle-income
Gambia	DHS 2019, DHS 2013, MICS 2010	African	Low-income
Ghana	DHS 2022, MICS 2011	African	Lower-middle-income
Guatemala	DHS 2015, RHS 2009	Americas	Upper-middle-income
Guinea	DHS 2018, DHS 2012, DHS 2005	African	Lower-middle-income
Guinea-Bissau	MICS 2019, MICS 2006	African	Low-income
Haiti	DHS 2016, DHS 2006	Americas	Lower-middle-income
Honduras	MICS 2019, DHS 2011	Americas	Lower-middle-income
India	DHS 2020, DHS 2006	South-East Asia	Lower-middle-income
Indonesia	DHS 2017, DHS 2007	Western Pacific	Upper-middle-income
Iraq	MICS 2018, MICS 2006	Eastern Mediterranean	Upper-middle-income
Jamaica	MICS 2022, MICS 2011	Americas	Upper-middle-income
Jordan	DHS 2023, DHS 2012	Eastern Mediterranean	Lower-middle-income
Kazakhstan	MICS 2015, MICS 2006	European	Upper-middle-income
Kenya	DHS 2022, DHS 2008	African	Lower-middle-income
Kiribati	MICS 2019	Western Pacific	Lower-middle-income
Kyrgyzstan	MICS 2023, DHS 2012	European	Lower-middle-income
Lao People's Democratic Republic	MICS 2017, MICS 2012, MICS 2006	Western Pacific	Lower-middle-income
Lesotho	DHS 2023, DHS 2009	African	Lower-middle-income
Liberia	DHS 2019, DHS 2013, DHS 2007	African	Low-income
Madagascar	DHS 2021, DHS 2008	African	Low-income
Malawi	MICS 2020, DHS 2010	African	Low-income

Maldives	DHS 2016, DHS 2009	South-East Asia	Upper-middle-income
Mali	DHS 2018, MICS 2010	African	Low-income
Mauritania	DHS 2020, MICS 2011	African	Lower-middle-income
Mexico	NSS 2019	Americas	Upper-middle-income
Mongolia	MICS 2018, MICS 2010	Western Pacific	Upper-middle-income
Mozambique	DHS 2022, DHS 2011	African	Low-income
Myanmar	DHS 2016	South-East Asia	Lower-middle-income
Nepal	DHS 2022, DHS 2011	South-East Asia	Lower-middle-income
Niger	DHS 2021, DHS 2012	African	Low-income
Nigeria	MICS 2021, DHS 2013, MICS 2011	African	Lower-middle-income
North Macedonia	MICS 2019, MICS 2011	European	Upper-middle-income
Pakistan	DHS 2017, DHS 2012, DHS 2006	Eastern Mediterranean	Lower-middle-income
Papua New Guinea	DHS 2017	Western Pacific	Lower-middle-income
Paraguay	MICS 2016, RHS 2008	Americas	Upper-middle-income
Peru	DHS 2023, DHS 2013	Americas	Upper-middle-income
Philippines	DHS 2022, DHS 2013	Western Pacific	Lower-middle-income
Rwanda	DHS 2019, DHS 2010	African	Low-income
Samoa	MICS 2020	Western Pacific	Upper-middle-income
Sao Tome and Principe	MICS 2019, DHS 2008	African	Lower-middle-income
Senegal	DHS 2023, DHS 2012	African	Lower-middle-income
Serbia	MICS 2019, MICS 2005	European	Upper-middle-income
Sierra Leone	DHS 2019, MICS 2010	African	Low-income
South Africa	DHS 2016	African	Upper-middle-income
Sudan	MICS 2014	Eastern Mediterranean	Low-income
Suriname	MICS 2018, MICS 2010, MICS 2006	Americas	Upper-middle-income

Tajikistan	DHS 2017, DHS 2012, MICS 2005	European	Lower-middle-income
Thailand	MICS 2022, MICS 2012	South-East Asia	Upper-middle-income
Timor-Leste	DHS 2016, DHS 2009	South-East Asia	Lower-middle-income
Togo	MICS 2017, MICS 2010, MICS 2006	African	Low-income
Tonga	MICS 2019	Western Pacific	Upper-middle-income
Tunisia	MICS 2018, MICS 2011	Eastern Mediterranean	Lower-middle-income
Turkmenistan	MICS 2015, MICS 2006	European	Upper-middle-income
Tuvalu	MICS 2020	Western Pacific	Upper-middle-income
Türkiye	DHS 2018, DHS 2008	European	Upper-middle-income
Uganda	DHS 2016, DHS 2006	African	Low-income
United Republic of Tanzania	DHS 2022, DHS 2010	African	Lower-middle-income
Uzbekistan	MICS 2021, MICS 2006	European	Lower-middle-income
Vanuatu	MICS 2023	Western Pacific	Lower-middle-income
Viet Nam	MICS 2020, MICS 2010	Western Pacific	Lower-middle-income
Yemen	MICS 2023, DHS 2013	Eastern Mediterranean	Low-income
Zambia	DHS 2018, DHS 2007	African	Lower-middle-income
Zimbabwe	MICS 2019, DHS 2010, MICS 2009	African	Lower-middle-income
occupied Palestinian territory	MICS 2019, MICS 2010	Eastern Mediterranean	Lower-middle-income

DHS: Demographic and Health Surveys; MICS: Multiple Indicator Cluster Surveys; NSS: non-standard national health surveys; RHS: Reproductive and Health Surveys

Table S2. Childhood immunization indicator median differences and ratios, by dimensions of inequality, 2014-2023 DHS, MICS and NSS

Indicator	Dimension	Number of countries	Median difference (95% CI) (percentage points)	Median ratio (95% CI)
Immunization coverage				
BCG	Child sex	91	0.07 (-0.24–0.32)	1.00 (1.00–1.00)
	Mother's age	42	0.96 (0.33–2.81)	1.01 (1.00–1.03)
	Mother's education	67	9.60 (6.03–14.26)	1.11 (1.07–1.18)
	Household economic status	90	4.32 (2.89–7.38)	1.05 (1.03–1.09)
	Place of residence	91	1.91 (1.03–2.78)	1.02 (1.01–1.03)
DTP3	Child sex	92	-0.03 (-0.71–0.48)	1.00 (0.99–1.01)
	Mother's age	42	2.43 (0.66–3.49)	1.03 (1.01–1.06)
	Mother's education	68	13.67 (10.42–19.55)	1.20 (1.15–1.36)
	Household economic status	91	7.97 (4.98–11.30)	1.11 (1.06–1.16)
	Place of residence	92	2.06 (0.69–3.66)	1.02 (1.01–1.04)
Measles	Child sex	92	0.16 (-0.42–0.71)	1.00 (0.99–1.01)
	Mother's age	42	3.22 (1.65–4.88)	1.04 (1.02–1.08)
	Mother's education	68	13.78 (12.60–19.25)	1.23 (1.18–1.32)
	Household economic status	91	9.03 (6.19–12.18)	1.13 (1.07–1.17)
	Place of residence	92	1.23 (-0.09–3.02)	1.01 (1.00–1.04)
Polio	Child sex	91	-0.05 (-0.73–0.43)	1.00 (0.99–1.00)
	Mother's age	42	2.68 (0.07–4.99)	1.04 (1.00–1.07)
	Mother's education	68	12.56 (8.89–16.88)	1.17 (1.13–1.34)
	Household economic status	90	7.38 (2.78–9.57)	1.09 (1.03–1.16)
	Place of residence	91	0.84 (-0.88–2.34)	1.01 (0.98–1.03)
Full	Child sex	90	0.03 (-0.81–0.87)	1.00 (0.99–1.01)
	Mother's age	42	2.89 (0.78–6.14)	1.05 (1.01–1.12)
	Mother's education	67	14.42 (10.61–18.68)	1.30 (1.22–1.46)

	Household economic status	89	7.42 (5.26–12.04)	1.14 (1.07–1.25)
	Place of residence	90	1.11 (-0.40–3.58)	1.02 (0.99–1.07)
Hib3	Child sex	90	-0.08 (-0.72–0.59)	1.00 (0.99–1.01)
	Mother's age	41	2.43 (0.14–4.03)	1.03 (1.00–1.07)
	Mother's education	66	13.67 (10.43–20.14)	1.20 (1.15–1.37)
	Household economic status	89	8.55 (4.51–11.47)	1.11 (1.05–1.17)
	Place of residence	90	2.28 (0.61–3.89)	1.03 (1.01–1.04)
Rotavirus	Child sex	54	0.17 (-0.68–0.52)	1.00 (0.99–1.01)
	Mother's age	30	2.26 (-0.17–4.01)	1.02 (1.00–1.06)
	Mother's education	43	9.64 (7.04–14.35)	1.13 (1.10–1.24)
	Household economic status	54	6.19 (3.90–11.64)	1.09 (1.05–1.16)
	Place of residence	54	1.43 (0.84–2.79)	1.02 (1.01–1.03)
Non-receipt of vaccines				
DTP (zero-dose)	Child sex	91	0.21 (-0.14–0.61)	1.04 (0.98–1.07)
	Mother's age	41	2.24 (-0.11–3.97)	1.27 (0.99–1.42)
	Mother's education	67	8.91 (6.53–16.17)	2.73 (2.34–3.12)
	Household economic status	90	4.34 (2.46–6.57)	2.16 (1.61–2.88)
	Place of residence	91	1.48 (0.69–3.20)	1.38 (1.15–1.54)
BCG, DTP, measles, and polio	Child sex	88	-0.02 (-0.13–0.14)	0.99 (0.94–1.03)
	Mother's age	41	0.34 (-0.31–1.50)	1.04 (0.85–1.51)
	Mother's education	66	5.97 (4.17–11.27)	3.23 (2.38–3.66)
	Household economic status	87	3.56 (2.33–4.63)	2.73 (2.03–3.83)
	Place of residence	88	1.24 (0.46–1.88)	1.55 (1.19–1.79)

BCG: Bacillus Calmette–Guérin; DHS: Demographic and Health Surveys; DTP3: three doses of diphtheria–tetanus–pertussis vaccine; Hib3: three doses of *Haemophilus influenzae* type b vaccine; MICS: Multiple Indicator Cluster Surveys; NSS: non-standard national health surveys. Polio indicator reflects the receipt of three doses of any polio vaccine; rotavirus indicator reflects the receipt of the last dose. Full includes BCG (one dose), DTP3, measles (one dose) and polio (three doses).

Table S3. Childhood immunization indicators (%), median differences by dimensions of inequality, 2004-2013 and 2014-2023 DHS, MICS, RHS and NSS

Indicator	Dimension	Number of countries	Median difference in time 0 (95% CI) (percentage points)	Median difference in time 1 (95% CI) (percentage points)	Median absolute change in difference over 10 years (95% CI) (percentage points)	Median SII in time 0 (95% CI) (percentage points)	Median SII in time 1 (95% CI) (percentage points)	Median absolute change in SII over 10 years (95% CI) (percentage points)
Immunization coverage								
BCG	Child sex	78	0.27 (-0.01–0.67)	0.14 (-0.12–0.57)	-0.25 (-0.76–0.15)			
	Mother's age	29	1.65 (-0.35–2.61)	1.29 (0.06–4.08)	0.10 (-1.85–1.50)			
	Mother's education	58	7.79 (4.89–15.64)	9.55 (6.05–14.24)	0.01 (-1.52–1.44)	11.24 (7.62–21.81)	10.45 (6.91–16.68)	-0.86 (-2.97–0.95)
	Household economic status	75	5.85 (2.06–8.48)	4.07 (2.64–7.72)	0.85 (-1.46–1.80)	7.68 (2.83–12.86)	5.22 (3.64–10.09)	0.06 (-1.80–1.59)
	Place of residence	78	3.15 (1.59–4.89)	1.93 (1.09–2.73)	-0.65 (-1.33–0.02)			
DTP3	Child sex	79	0.35 (-0.09–0.72)	-0.09 (-0.69–0.47)	-0.23 (-1.36–0.88)			
	Mother's age	29	1.89 (0.80–4.08)	1.78 (-0.38–2.51)	-0.96 (-2.57–3.25)			
	Mother's education	59	17.94 (10.75–23.53)	13.62 (10.42–18.83)	-3.63 (-7.00–0.63)	21.45 (14.96–28.49)	17.68 (12.36–23.27)	-3.66 (-7.77–-0.36)
	Household economic status	76	9.57 (4.41–17.13)	8.11 (4.92–11.27)	-3.25 (-6.10–-0.77)	13.03 (4.76–20.12)	9.96 (5.27–13.44)	-3.44 (-6.22–-0.51)
	Place of residence	79	2.77 (1.48–7.14)	1.61 (0.42–3.47)	-2.42 (-3.70–-0.77)			
Measles	Child sex	79	0.00 (-0.70–0.48)	0.40 (-0.37–0.86)	0.77 (-0.31–1.41)			
	Mother's age	29	0.12 (-0.84–3.41)	1.95 (1.16–4.66)	1.48 (-1.78–3.77)			
	Mother's education	59	17.61 (13.74–20.48)	13.62 (12.05–17.57)	-2.10 (-5.87–3.83)	21.99 (17.67–25.86)	18.34 (17.07–24.12)	-0.16 (-2.66–3.77)
	Household economic status	76	10.79 (6.49–15.12)	9.41 (5.82–12.54)	-1.35 (-3.26–0.88)	12.39 (7.47–18.17)	11.39 (6.51–15.60)	-1.66 (-4.20–0.39)
	Place of residence	79	4.92 (3.61–6.16)	1.78 (0.17–3.54)	-2.16 (-3.93–-1.38)			
Polio	Child sex	78	0.20 (-0.33–0.85)	-0.03 (-0.72–0.39)	-0.64 (-1.68–0.46)			
	Mother's age	29	3.10 (0.52–5.12)	2.13 (-0.57–3.89)	-2.44 (-4.26–1.93)			
	Mother's education	59	11.30 (9.63–15.78)	12.45 (8.90–15.36)	-0.24 (-3.07–2.84)	17.28 (11.99–21.56)	15.61 (11.51–20.68)	-1.78 (-4.37–2.39)

	Household economic status	76	8.51 (5.40–15.21)	6.86 (1.60–9.53)	-2.66 (-4.93–0.16)	10.12 (5.94–17.19)	8.27 (2.77–11.14)	-3.22 (-5.85–-0.28)
	Place of residence	78	1.84 (0.44–5.52)	0.79 (-1.53–1.89)	-1.80 (-3.89–0.91)			
Full	Child sex	77	-0.03 (-0.79–0.54)	0.01 (-0.79–0.87)	-0.06 (-1.59–0.76)			
	Mother's age	29	2.44 (0.23–3.89)	2.42 (0.58–6.94)	-0.77 (-3.55–4.40)			
	Mother's education	58	16.92 (13.42–20.42)	14.26 (10.62–18.68)	-1.22 (-6.18–3.65)	21.73 (16.80–26.81)	17.61 (14.57–24.66)	-1.62 (-6.33–3.19)
	Household economic status	75	10.57 (5.73–17.91)	7.42 (4.17–11.97)	-3.28 (-5.74–0.39)	12.99 (6.51–21.82)	9.97 (5.20–14.18)	-4.31 (-8.07–0.86)
	Place of residence	77	4.60 (0.27–6.12)	1.28 (-0.56–3.64)	-2.78 (-5.58–1.00)			
Hib3	Child sex	50	0.35 (-0.25–0.71)	-0.14 (-0.82–0.71)	-0.23 (-2.43–1.29)			
	Mother's age	20	1.84 (0.50–4.71)	2.05 (-0.66–5.38)	-0.48 (-5.55–4.13)			
	Mother's education	40	11.85 (7.87–20.40)	12.69 (9.68–17.62)	0.66 (-7.49–4.54)	15.11 (10.55–19.17)	15.81 (11.22–22.79)	-1.43 (-9.84–6.20)
	Household economic status	48	10.52 (2.80–13.33)	8.39 (4.11–11.57)	-2.90 (-6.14–0.10)	10.91 (4.82–17.17)	9.97 (4.97–12.70)	-2.77 (-6.25–1.05)
	Place of residence	50	1.89 (0.77–5.05)	1.32 (0.27–3.25)	-2.01 (-4.69–1.63)			
Non-receipt of vaccines								
DTP (zero-dose)	Child sex	74	0.37 (-0.19–0.88)	0.31 (-0.04–0.62)	-0.47 (-0.88–0.24)			
	Mother's age	28	-0.15 (-1.11–1.36)	0.54 (-0.71–3.73)	0.03 (-0.85–2.80)			
	Mother's education	56	9.16 (6.74–16.18)	8.94 (6.58–16.10)	-0.39 (-1.83–1.33)	-13.54 (-21.93–-9.02)	-11.40 (-19.97–-7.97)	1.25 (-1.87–2.25)
	Household economic status	71	6.54 (3.79–10.11)	4.63 (2.31–6.95)	-0.77 (-2.75–0.88)	-8.03 (-13.03–-4.05)	-6.74 (-8.48–-3.52)	1.01 (-0.29–3.24)
	Place of residence	74	3.14 (1.60–4.68)	1.68 (0.62–3.59)	-0.75 (-1.40–0.04)			
BCG, DTP, measles, and polio	Child sex	69	0.20 (-0.15–0.51)	0.03 (-0.11–0.18)	-0.39 (-0.70–0.14)			
	Mother's age	28	0.92 (-0.06–1.71)	0.30 (-0.43–2.77)	-0.09 (-1.40–1.20)			
	Mother's education	55	5.20 (3.25–7.08)	6.19 (4.27–11.75)	0.59 (-0.19–2.26)	-7.62 (-13.44–-5.76)	-8.05 (-14.09–-5.61)	-0.08 (-2.22–2.02)
	Household economic status	67	4.16 (2.00–6.94)	3.56 (2.31–5.13)	0.16 (-0.72–1.11)	-5.93 (-9.92–-2.66)	-4.25 (-7.30–-2.46)	0.56 (-1.12–1.81)

Place of residence	69	2.12 (1.14–3.08)	1.35 (0.54–2.39)	-0.59 (-1.00–0.09)

BCG: Bacillus Calmette–Guérin; DHS: Demographic and Health Surveys; DTP3: three doses of diphtheria–tetanus–pertussis vaccine; Hib3: three doses of *Haemophilus influenzae* type b vaccine; MICS: Multiple Indicator Cluster Surveys; NSS: non-standard national health surveys; RHS: Reproductive and Health Surveys. Polio indicator reflects the receipt of three doses of any polio vaccine; rotavirus indicator reflects the receipt of the last dose. Full includes BCG (one dose), DTP3, measles (one dose) and polio (three doses). Time 0 represents the date range 2004-2013 and time 1 represents the data range 2014-2023. For each country, the absolute change in difference (over 10 years) was calculated as the most recent difference (time 1) minus the prior difference (time 0), divided by the number of intervening years, and multiplied by 10.
